# Induction of Sodium/Iodide Symporter (NIS) Expression and Radioiodine Uptake in Non-Thyroid Cancer Cells

**DOI:** 10.1371/journal.pone.0031729

**Published:** 2012-02-16

**Authors:** Zhi Liu, Mingzhao Xing

**Affiliations:** Laboratory for Cellular and Molecular Thyroid Research, Division of Endocrinology and Metabolism, School of Medicine, Johns Hopkins University, Baltimore, Maryland, United States of America; Cardiff University, United Kingdom

## Abstract

**Background:**

This study was designed to explore the therapeutic potential of suppressing MAP kinase and PI3K/Akt pathways and histone deacetylase (HDAC) to induce the expression of sodium/iodide symporter (NIS) and radioiodine uptake in non-thyroid cancer cells.

**Methods:**

We tested the effects of the MEK inhibitor RDEA119, the Akt inhibitor perifosine, and the HDAC inhibitor SAHA on NIS expression in thirteen human cancer cell lines derived from melanoma, hepatic carcinoma, gastric carcinoma, colon carcinoma, breast carcinoma, and brain cancers. We also examined radioiodine uptake and histone acetylation at the NIS promoter in selected cells.

**Results:**

Overall, the three inhibitors could induce NIS expression, to various extents, in melanoma and all the epithelial carcinoma-derived cells but not in brain cancer-derived cells. SAHA was most effective and its effect could be significantly enhanced by RDEA119 and perifosine. The expression of NIS, at both mRNA and protein levels, was most robust in the melanoma cell M14, hepatic carcinoma cell HepG2, and the gastric carcinoma cell MKN-7 cell. Radioiodine uptake was correspondingly induced, accompanied by robust increase in histone acetylation at the NIS promoter, in these cells when treated with the three inhibitors.

**Conclusions:**

This is the first demonstration that simultaneously suppressing the MAP kinase and PI3K/Akt pathways and HDAC could induce robust NIS expression and radioiodine uptake in certain non-thyroid human cancer cells, providing novel therapeutic implications for adjunct radioiodine treatment of these cancers.

## Introduction

As a transmembrane glycoprotein expressed primarily in follicular epithelial thyroid cells, sodium iodide symporter (NIS) plays a fundamental role in the transportation of iodide from the extracellular space into the thyroid cell for synthesis of thyroid hormones in the thyroid gland [1]–[4]. This is the biological basis for the clinical application of radioiodine in the diagnosis and treatment of a variety of benign and malignant thyroid diseases. A typical example of utilizing this function of NIS is the radioiodine ablation widely applied for the treatment of thyroid cancer [5], [6]. Radioiodine treatment is usually effective in thyroid cancer patients, but it becomes ineffective when thyroid cancer cells have lost the expression of NIS and can no longer take up radioiodine as typically seen in poorly differentiated and undifferentiated thyroid cancers [7]–[10]. Previous studies demonstrated that inhibitors of histone deacetylase (HDAC) could induce the expression of NIS in thyroid cancer cells [11], [12]. We recently demonstrated that combination of the HDAC inhibitor SAHA with inhibitors of the MAP kinase and PI3K/Akt pathways could induce robust and synergistic expression of NIS and radioiodine uptake in thyroid cancer cells [13]. This opened the possibility for novel effective treatment of thyroid cancer using radioiodine that is otherwise non-avid for this radioisotope.

Given the unique function of NIS to transport iodide into thyroid cells and the clinical success of using radioiodine for thyroid cancer ablation treatment, it has long been proposed and tested that exogenously induced NIS expression using targeted gene transfer can confer non-thyroid cancer cells radioiodine avidity for radioiodine ablation treatment [2]–[4], [14]. Such studies are promising but have not yet resulted in reliable clinical applications. Major effort is still needed to improve several key aspects of this approach, including the therapeutic efficacy, specificity, safety, and technical complexity. NIS can be expressed in various normal non-thyroid tissues, although at a low level, including, for example, salivary, lacrimal, breast, stomach, intestine, lung, and kidney tissues [15]–[19]. Low-level expression of NIS was also reported in some non-thyroid cancers such as breast carcinoma [20]. We recently demonstrated that suppression of the MAP kinase and PI3K/Akt pathways could induce expression of NIS and radioiodine uptake in melanoma cells [21]. Given the synergism in robustly inducing thyroid gene expression and radioiodine uptake in thyroid cancer cells by simultaneously inhibiting HDAC and the MAP kinase and PI3K/Akt pathways [13], in the present study we tested the potential of this novel therapeutic strategy to induce NIS expression for radioiodine uptake in an extended panel of non-thyroid cancer cells.

## Results

### Induction of NIS gene expression in non-thyroid cancer cells by suppressing the MAP kinase and PI3K/AKT pathways and HDAC

**Figure 1:**
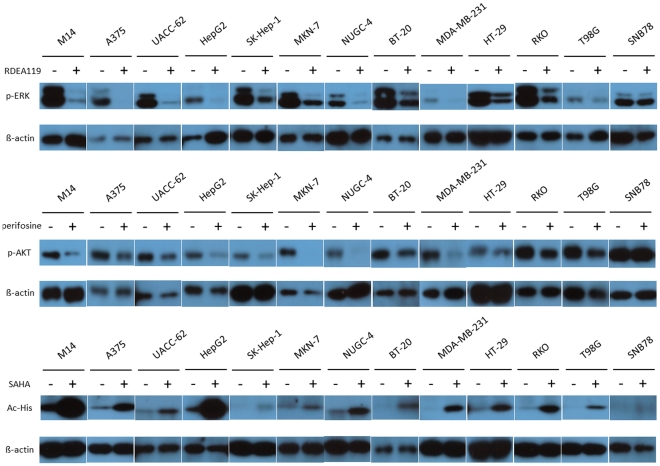
Target effects of inhibitors of the MAP kinase and PI3K/Akt pathways and HDAC in various non-thyroid human cancer cells. The MEK inhibitor RDEA119, the Akt inhibitor perifosine, and the HDAC inhibitor SAHA were used to respectively target these signaling pathways or molecules. Cells were treated for 30 hrs with 0.5 µM RDEA119, 5 µM perifosine or 0.5 µM SAHA as indicated. DMSO or PBS was used in parallel as the vehicle control. Cells were lysed for Western blotting after treatments to reveal the levels of phosphorylated ERK (p-ERK), phosphorylated Akt (p-Akt), and acetylated histone (Ac-His) with specific antibodies. “+”, treatment with the indicated inhibitor; “−”, treatment with vehicle.

We tested the effects of the MEK inhibitor RDEA119, the Akt inhibitor perifosine, and the HDAC inhibitor SAHA on the expression of the *NIS* gene in 13 human cancer cells (Table 1). These included melanoma cells and epithelial carcinoma cells derived from hepatocarcinoma, gastric carcinoma, colon carcinoma, and breast cancer, as well as glioblastoma cell T98G and astrocytoma cell SNB-78. To demonstrate the targeted drug effects of the three inhibitors in these cells, we first tested their effects on their target molecules in the signaling pathways. As shown in Fig. 1, after 30 hrs of treatment, RDEA119 at 0.5 µM and perifosine at 5 µM could significantly inhibit the phosphorylation of ERK (p-ERK) and the phosphorylation of AKT (p-AKT), respectively, in virtually all the cells except for the SNB-78 cell. SAHA at 0.5 µM for 30 hrs could dramatically enhance the acetylation of histone H3 in these cells except for few cells, such as T98G and SNB78, in which there was no or only a small increase in histone acetylation. These results, overall, demonstrated the expected target effects of these inhibitors.

**Table 1:** NIS mRNA expression in different human cancer cells after drug treatments (mean±SD).*

		RD	PE	SA	RD+PE	RD+SA	PE+SA	RD+PE+SA
Melanoma	**M14**	**13.3±2.9**	**2.7±0.5**	**145.6±1.1**	**11.2±2.5**	**554.2±15.5**	**225.6±16.7**	**661.3±19.3**
	A375	5.3±1.4	1.0±0.1	6.1±3.8	4.1±1.3	8.9±1.9	4.6±3.1	6.2±1.5
	UACC-62	0.7±0.1	2.0±0.8	4.8±0.8	0.5±0.2	3.9±1.3	18.3±4.9	6.6±4.8
Liver	**HepG2**	**3.1±0.9**	**1.2±0.2**	**64.1±15.8**	**3.2±0.5**	**77.5±1.9**	**64.7±9.3**	**74.4±29.4**
	SK-Hep-1	1.9±0.5	1.7±0.4	6.9±2.1	1.7±0.4	8.1±2.5	8.0±0.6	9.8±2.8
Gastric	**MKN-7**	**6.0±0.1**	**1.5±0.3**	**11.5±0.1**	**4.5±1.3**	**19.4±3.2**	**18.5±5.2**	**25.1±4.2**
	NUGC-4	1.1±0.5	1.6±1.3	6.7±2.1	0.8±0.5	8.2±0.1	6.2±0.4	5.5±0.6
Breast	MDA-MB-231	2.4±1.0	0.4±0.1	1.3±0.1	2.4±0.6	5.3±2.6	2.0±0.3	3.6±0.9
	BT-20	2.3±1.3	1.2±1.0	7.2±5.0	2.6±1.9	4.2±2.7	4.8±2.2	5.2±2.7
Colon	RKO	2.1±0.1	1.5±0.6	2.3±0.9	3.3±2.0	3.1±1.2	2.2±0.8	4.0±1.1
	HT-29	3.1±1.0	0.6±0.3	1.3±0.9	3.8±1.6	5.5±0.9	0.9±0.7	6.0±2.3
Non-epithelial	T98G	1.3±0.7	0.7±0.2	1.8±0.4	0.6±0.3	1.7±0.6	1.7±0.5	1.4±0.1
	SNB78	0.8±0.2	0.6±0.1	0.7±0.1	1.1±0.4	0.9±0.2	1.1±0.1	1.1±0.3

*The data in the table represent folds of increase in NIS expression over the control treatment with DMSO/PBS as described in the Materials and Methods. RD, RDEA119; PE, perifosine; SA, SAHA.

We subsequently tested the effects of the three inhibitors at these concentrations, individually or in combinations, on the expression of the *NIS* gene in the 13 cells using quantitative real-time PCR. As shown in Table 1, *NIS* expression was increased to various extents in all the cells, except for the two non-epithelial cancer cells T98G and SNB78 which did not have any response to these drug treatments. The induced *NIS* expression was most robust in the melanoma cell M14, the hepatocarcinoma cell HepG2, and the gastric carcinoma cell MKN-7, which were used for further studies as presented in the following sections. Individually, RDEA119 and perifosine each had a smaller effect and SAHA displayed the most pronounced effects on the expression of *NIS*. A synergistic effect was seen when the MEK inhibitor or the Akt inhibitor was each combined with SAHA and the synergistic effects were even more robust when the three drugs were combined, with an exception that in HepG2 cells perifosine did not enhance further the robust effect of SAHA.

**Figure 2:**
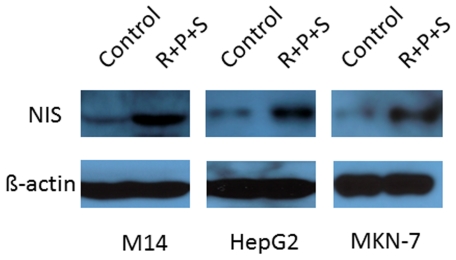
Western blotting analysis of NIS protein expression in M14, HepG2 and MKN-7 cells after treatment with the MEK, Akt and HDAC inhibitors. Cells were treated for 30 hrs with combination use of RDEA119, perifosine, and SAHA at the concentrations described in Fig. 1, followed by standard Western blotting analysis of cell lysates using the specific primary antibody against NIS. The expression level of ß-actin was analyzed in parallel for quality control. “Control”, treatment of cells with vehicle; “R+P+S”, treatment of cells with combined use of RDEA119, perifosine and SAHA.

Following the demonstration of mRNA expression of NIS by quantitative real-time PCR in melanoma and epithelial carcinoma cells, we next sought to examine the expression of NIS at the protein level in these cells, using the melanoma cell M14, hepatocarcinoma cell HepG2, and gastric carcinoma cell MKN-7 as representatives which displayed the most robust expression of NIS (Table 1). As shown in Fig. 2, combination treatment of these cells with RDEA119, perifosine and SAHA significantly increased NIS protein expression as detected by Western blotting. Compared with M14 and HepG2 cells, MKN-7 cells displayed a modest increase in the expression of NIS protein in response to the combination treatment with the three inhibitors, which was similar to the pattern of NIS mRNA expression in these cells under this drug treatment condition (Table 1). Thus, suppressing the MAP kinase and PI3K/Akt pathways and HDAC could induce robust expression of NIS in these cells both at the transcriptional and translational levels.

### Cellular radioiodide uptake induced by suppressing the MAP kinase and PI3K/Akt pathways and HDAC

**Figure 3:**
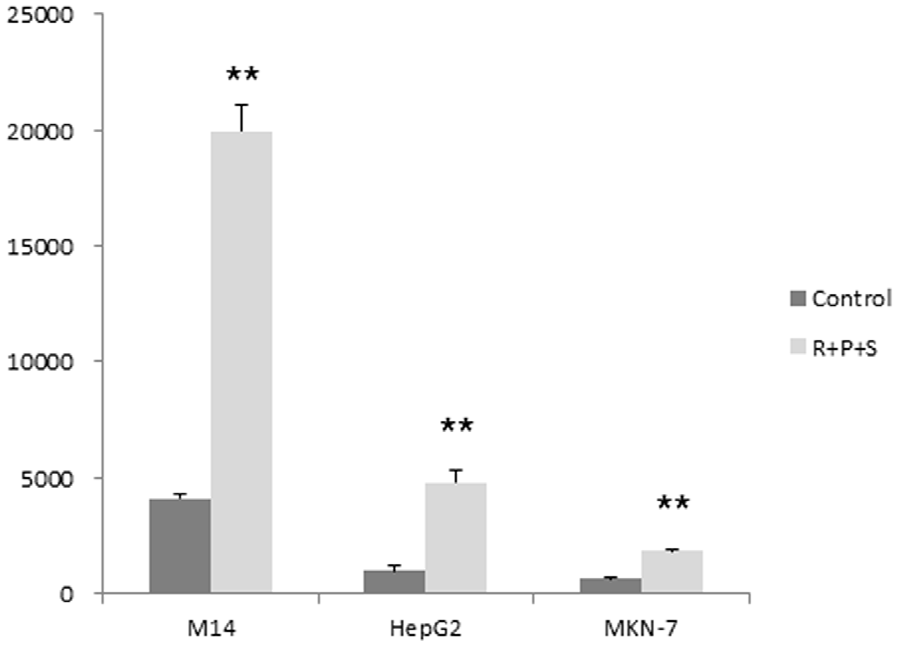
Induction of radioiodine uptake in M14, HepG2 and MKN-7 cells by treatment with the MEK, Akt and HDAC inhibitors. Cells were treated with RDEA119, perifosine and SAHA as described in Fig. 2, followed by incubation with Na^125^I for 1 hr. Parallel cells were additionally treated with the NIS blocker NaClO_4_ to obtain non-specific radioiodine uptake/binding with the cells. Cells were then washed, lysed, and measured for radioactivity. Cell radioiodine uptake is presented as cpm/10^6^ cells (on the y-axis of the figure) after correction for the non-specific radioiodine binding. Detailed experimental procedures are as described in the Materials and Methods. “Control”, treatment of cells with vehicle; “R+P+S”, treatment of cells with combination use of RDEA119, perifosine and SAHA. ** In comparison with control, p<0.01.

With the demonstration of robust NIS expression in non-thyroid cancer cells by suppressing the MAP kinase and PI3K/Akt pathways and HDAC, we used M14, HepG2 and MKN-7 cells to test the ultimate functional relevance of this finding by examining the ability of these cells to take up radioiodide after induction of NIS. As shown in Fig. 3, radioiodide uptake was clearly induced in these cells by the combination treatment with RDEA119, perifosine and SAHA. Among the three cells, marked radioiodide uptake was particularly seen in the M14 cell, consistent with the finding that NIS expression at both mRNA and protein levels was most robust in this cell.

### Enhancement of histone acetylation of the NIS promoter by suppressing the MAP kinase and PI3K/Akt pathways and HDAC

**Figure 4:**
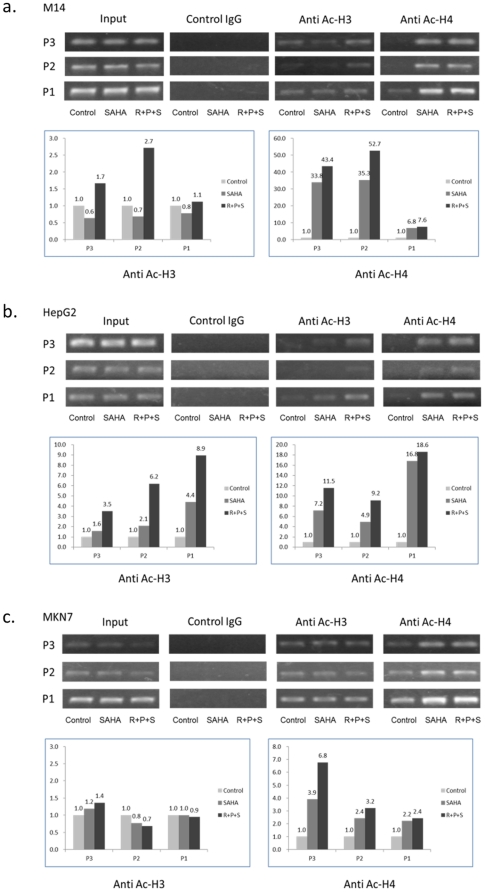
Effects of the treatment with MEK, Akt and HDAC inhibitors on histone acetylation at the NIS promoter in M14, HepG2 and MKN-7 cells. Cells were treated with SAHA alone or SAHA in combination with RDEA119 and perifosine as described in Fig. 2. Histone acetylation levels at the three regions (P1, P2, and P3) that comprise the minimal essential promoter of the *NIS* gene were analyzed by chromatin immunoprecipitation (ChIP) analysis as described in Materials and Methods. Acetylation status of both histone H3 and H4 was examined using specific antibodies for ChIP. Non-specific IgG antibodies were used as control. The results for M14, HepG2 and MKN-7 cells are presented in Figs. 4a, 4b and 4c, respectively. For each cell, the upper panel shows the actual results of PCR on the DNA fragments obtained by ChIP using non-specific control IgG, anti-acetylated H3 (Anti Ac-H3) antibody or anti-acetylated H4 (Anti Ac-H4) antibody. Identical amounts of pre-immunoprecipitation cell lysates from different treatment conditions were used to start the immunoprecipitation. An aliquot of pre-immunoprecipitation cell lysate was directly used to isolate DNA as “Input” control. The PCR results reflect the histone acetylation levels. Presented in the lower panel for each cell is a bar graph showing quantitatively the acetylation levels of H3 and H4 at the three regions of *NIS* promoter based on densitometric measurements of the upper panel. The results are normalized by dividing the corresponding input signals and are presented as the ratio of the indicated treatment over the control. “Control”, treatment of cells with vehicle; “SAHA”, treatment of cells with SAHA alone; “R+P+S”, treatment of cells with combined use of RDEA119, perifosine and SAHA.

Histone acetylation at the promoter region promotes gene expression [22]. To explore whether this was a mechanism involved in the expression of NIS induced by the drug treatments in non-thyroid cancer cells in the present study, we next performed ChIP analysis of histone acetylation at the NIS promoter. We examined three different regions of the NIS promoter for histone H3 and H4 acetylation in M14, HepG2 and MKN-7 cells. These regions represent the minimal essential NIS promoter [23]. We found that histone acetylation at these regions of the NIS promoter was increased to various levels by treatment with SAHA in these cells (Fig. 4a–c). Combination treatment with the MAP kinase and PI3K/Akt pathways and HDAC inhibitors increased further histone acetylation at the NIS promoter. Overall, this histone acetylation was seen in both H3 and H4 in the three cells except for MKN-7 cells in which no significant acetylation change was seen in H3 (Fig. 4c). All the three regions of the minimal essential NIS promoter displayed histone acetylation except for the P1 region in M14 cells that showed no change in acetylation of H3 after drug treatments (Fig. 4a). Up to about 50 folds of increase in histone H4 acetylation at the P2 region of the NIS promoter in M14 cells (Fig. 4a) and about 20 folds of increase in histone H4 acetylation at the P1 region in HepG2 cells (Fig. 4b) were observed. These data suggest that histone acetylation is a mechanism involved in the NIS expression induced by suppressing the MAP kinase and PI3K/Akt pathways and HDAC in these non-thyroid cancer cells. M14 cells showed the most robust histone acetylation at the NIS promoter and second most in HepG2 cells. MKN-7 cells displayed a relatively modest histone acetylation at the NIS promoter. It is interesting to note that this pattern of the extent of histone acetylation in the three cells echoed well the patterns of the extent of NIS expression (Table 1, Fig. 2) and radioiodine uptake (Fig. 3) in these cells, further supporting an important role of histone acetylation in the NIS expression induced by targeting the MAP kinase and PI3K/Akt pathways and HDAC. This does not seem to apply to the T98G cell which showed some histone acetylation under the treatment with SAHA (Fig. 1) while there was no increase in NIS expression in this cell (Table 1). However, this acetylation level of histone was minimal in comparison with that in many other cells (Fig. 1). Therefore, the correlation of NIS expression with histone acetylation was overall clear in various cells tested.

## Discussion

Radioiodine ablation and adjuvant therapy are classical and standard treatments for thyroid cancer, which takes advantage of the unique iodide-transporting function of NIS in the thyroid cell membrane. Given the fact that NIS is also normally expressed, to various extents, in several non-thyroid tissues, in the present study we tested *NIS* expression and radioiodine uptake in cancer cells derived from non-thyroid tissues by simultaneously suppressing the MAP kinase and PI3K/Akt pathways and HDAC. Although the role of MAP kinase and PI3K/Akt pathways in the expression of NIS was investigated in melanoma in our previous studies [21], the role of HDAC and histone acetylation in the expression of *NIS* in melanoma has not been investigated. We therefore also included melanoma cells in the present study.

We were able to induce expression of NIS, to various extents, in melanoma cells and hepatic, gastric, colon and breast carcinoma cells, but not in non-epithelial brain tumor-derived cells, suggesting that this inducible NIS expression may be restricted to skin cancer cells and cancer cells of epithelial cell origin. This is interestingly consistent with the distribution pattern of physiological uptake of radioiodine in the tissues of skin (recovery phase of burn), liver, stomach, colon, and breast (lactating phase) on the body scan of thyroid cancer patients after radioiodine treatment [24]–[27]. Remarkably, we also demonstrated radioiodine uptake following the induced expression of NIS, consistent with NIS functionality in these non-thyroid cancer cells. Our data also for the first time showed that increased histone acetylation at the *NIS* promoter was an important mechanism involved in the expression of NIS in these non-thyroid cancer cells.

HDAC inhibitor-induced NIS expression was previously demonstrated in Hela cells and, as in the same study, in HepG2 cells [28]. To take a further step, we recently demonstrated a synergistic effect of the HDAC inhibitor SAHA on NIS expression induced by suppressing the MAP kinase and PI3K/Akt pathways in thyroid cancer cells although, in this study, histone acetylation was not examined [13]. In the present study, we for the first time directly demonstrated that suppression of the MAP kinase and PI3K/Akt pathways enhanced histone acetylation at the *NIS* promoter as a mechanism for the expression of NIS induced by simultaneously targeting the two pathways and HDAC in human cancer cells.

Demonstration of NIS expression at both mRNA and protein levels as well as radioiodide uptake in these cells established the functional significance of the inducible expression of the *NIS* gene in these non-thyroid cancer cells. The level of induced NIS expression varied among the cells tested, with melanoma cell M14, hepatic carcinoma cell HepG2, and gastric carcinoma cell MKN-7 showing the most robust expression of NIS, suggesting that NIS expression and radioiodine uptake can be induced preferentially in certain specific cases of these cancers for unclear reasons. Additional factors likely exist that determine the inducibility of NIS expression in these cancer cells, as exemplified by the estrogen receptor status that determines the inducibility of NIS expression by all-trans retinoic acid in breast cancer cells [29]. The results of the present study have important clinical implications. Melanoma, hepatic carcinoma, and gastric carcinoma are common human cancers, all with high mortality rates, particularly in metastatic and advanced cases. As the latter cases are usually surgically inoperable, novel effective medical treatments are currently urgently needed. With the inducibility of NIS expression and radioiodine uptake in these cancer cells demonstrated in the present study, it may be possible to treat these cancers using adjunct radioiodine as in the treatment for thyroid cancer. The fact that induced NIS expression was particularly robust in some cell lines but not others derived from these cancers suggests that this radioiodine treatment may be effective in a subgroup of patients with these cancers.

Therapeutic agents targeting major signaling pathways, such as MEK and BRAF inhibitors for the MAP kinase pathway and Akt and mTOR inhibitors for the PI3K/Akt pathway, as well as HDAC inhibitors, are currently being actively developed clinically [30]–[33]. Many of them have shown strong therapeutic potential in various human cancers and some have been approved for clinical use. It is therefore clinically possible to use these inhibitors to target the MAP kinase and PI3K/Akt pathways and some of them in combination to dually suppress the two pathways to induce NIS expression. The HDAC inhibitor SAHA, which has also been approved for the treatment of certain cancers [34], can be included in this drug combination therapy to enhance NIS expression for radioiodine treatment of cancers. As these drugs are themselves therapeutic by directly inhibiting cancer cell growth, their use in conjunction with radioiodine use to further kill cancer cells could make the treatment an even more effective chemotherapy. This may prove to be a novel and particularly effective therapeutic strategy for melanoma and carcinomas of the hepatic and gastrointestinal system. This technically easy strategy of using clinically proven drugs to induce NIS expression and radioiodine uptake in non-thyroid cancer cells, if confirmed to be effective in future studies, might prove to be superior to NIS expression achieved by gene transfer approaches as it can avoid certain safety, specificity, efficacy, and technical complexity issues associated with the widely investigated plasmid- or virus-mediated organ-targeted NIS gene transfer [4], [14].

Interestingly, previous studies demonstrated a basal expression of thyroid genes in skin cells and melanoma cells [35], [36]. This provides a basis for the robustly induced expression of thyroid genes and radioiodine uptake in melanoma cells by targeting the MAP kinase and PI3K/Akt pathways and HDAC in our present and previous studies [21]. It is also possible but remains to be investigated that a low basal expression state of thyroid genes similarly exists in hepatic and gastric carcinomas and other cancers examined in the present study that becomes robustly active upon suppression of the major signaling pathways.

In summary, the present study for the first time demonstrates robust NIS expression and radioiodine uptake in several non-thyroid cancer cells by simultaneously targeting the MAP kinase and PI3K/Akt pathways and HDAC using their inhibitors. As many of these inhibitors have already been proven to be clinically feasible and promising in inhibiting various cancers, their use to also induce NIS expression for adjuvant radioiodine treatment in addition to their direct inhibition of cancer cells may prove to be a novel and effective therapeutic strategy for selected non-thyroid cancers.

## Materials and Methods

### Human cancer cell lines

Thirteen human cancer cell lines were used in this study, including melanoma cells M14, UACC-62 and A375; hepatic carcinoma cells HepG2 and SK-Hep-1; gastric carcinoma cells MKN-7 and NUGC-4; colon carcinoma cells HT-29 and RKO; breast carcinoma cells BT-20 and MDA-MB-231; glioblastoma cell T98G; and astrocytoma cell SNB-78. These cells were from American Type Culture Collection (Manassas, VA) and NCI-Frederick Cancer DCTD Tumor/Cell Line Repository (Frederick, MD). M14, UACC-62, MKN-7, NUGC-4 and SNB-78 cells were cultured in RPMI 1640 medium (Mediatech, Inc., Manassas, VA) with 10% fetal bovine serum (Invitrogen Corp. Carlsbad, CA). RKO, BT-20, MDA-MB-231, HepG2, SK-Hep-1, and T98G cells were cultured in Eagle's Minimum Essential Medium (ATCC, Manassas, VA) with 10% fetal bovine serum. The HT-29 cells were cultured in McCoy's 5a Medium (ATCC, Manassas, VA) with 10% fetal bovine serum. The A375 cells were cultured in Dulbecco's Modified Eagle's Medium (ATCC, Manassas, VA) with 10% fetal bovine serum. All the cells were grown at 37°C with 5% CO_2_.

Under these culture conditions, cells were treated, where indicated, with the MEK inhibitor RDEA119, the Akt inhibitor perifosine, and the HDAC inhibitor SAHA individually or in combinations. DMSO and PBS were used in parallel as the vehicle control.

### Western blotting

Cells were lysed in RIPA buffer with standard protease inhibitors (Santa Cruz Biotechnology, Santa Cruz, CA) and standard Western blotting analyses were performed as described previously [37] using primary antibodies, including anti-phospho-ERK, anti-phospho-Akt, anti-Ac-Histone H3, or anti-actin (Santa Cruz Biotechnology, Santa Cruz, CA); anti-Ac-Histone H4 (Cell Signaling, Danvers, MA); or anti-NIS antibodies (Millipore Corp., Billerica, MA).

### RNA extraction and quantitative real-time PCR

After a 30-h treatment of cells with the indicated inhibitors, total RNA was isolated using the TRIzol reagent (Invitrogen, Carlsbad, CA) following instructions of the manufacturer. Three µg total RNA was reverse-transcribed using Oligo-dT primers and superscript III RT (Invitrogen, Carlsbad, CA) according to the manufacturer's instructions. Quantitative real-time PCR was preformed to analyze the expression of the *NIS* gene using iTaq SYBR Green Supermix with ROX (Bio-rad, Hercules, CA) on a ABI 7900HT PCR system (Applied Biosystems, Foster City, CA). β-actin was run in parallel to standardize the input cDNA. The primers designed for *NIS* and *β-actin* genes and the methods used to calculate relative expression levels of *NIS* were as described previously [13].

### Radioactive iodide uptake assay

The radioactive iodide uptake assay was performed as described previously [13]. Briefly, after a 30-h treatment with the indicated inhibitors, cells were incubated with 1 µCi Na^125^I and 5 µM nonradioactive NaI in 2 ml medium on 6-well plates at 37°C for 1 hr. To determine the NIS-specific uptake, parallel cells similarly treated with the inhibitors were additionally treated with 200 µM NaClO_4_ for 30 min prior to Na^125^I treatment. The medium was aspirated and cells were quickly washed three times with Hank's balanced salt solution (Sigma-Aldrich, St. Louis, MO). Cells were then trypsinized and lysed with 1 ml 0.33 M NaOH. Parallel plates of cells identically treated with the inhibitors but without radioiodine incubation were used to determine the number of cells at the end of the experiment. The radioactivity associated with cells was counted with a gamma counter and presented as cpm/10^6^ cells after correction for non-specific radioiodide binding of cells in the presence of NaClO_4_.

### Chromatin immunoprecipitation (ChIP) assay

ChIP assay was performed using the EZ-Magna ChIP A kit (Millipore Corp., Billerica, MA) according to the manufacturer's protocol. Briefly, after drug treatments, 1×10^7^ cells were cross-linked with 37% formaldehyde for 10 min, followed by incubation with the glycine solution provided in the kit for 5 min. After 2 washings with PBS, cells were lysed and sonicated 12 times for 10 seconds at 20% pulse power using the Sonifier Cell Disrupter Model Micros-150D (Branson, Danbury, CT). The cross-linked protein/DNA was subsequently incubated overnight with anti-acetylated histone H3, anti-acetylated histone H4 antibodies or control non-specific IgG, and purified using protein A beads. After washing with the buffers in the kit in the order per the provided protocol, protein A beads with protein/DNA complexes were incubated with proteinase K at 62°C for 2 hours with shaking. DNA fragments were separated subsequently by centrifugation and purified using spin columns. Three pairs of primers spanning the minimal essential promoter area of the NIS gene were used in the analysis as described previously [23]. Standard end-point PCR was performed as follows: after 3 min denaturing at 95°C, the reaction mixture was run for 32 cycles at 94°C, 60°C, and 72C° each for 30 sec, followed by an elongation at 72°C for 8 min (for all three pairs of primers).

### Statistical Analysis

The data presented here are representatives of at least two similar experiments performed in duplicates or triplicates. The differences between groups were analyzed by *t* test and a *P* value less than 0.05 was considered significant.
